# Ferromagnetic Ground States in Face-Centered Cubic Hubbard Clusters

**DOI:** 10.1371/journal.pone.0161549

**Published:** 2016-09-01

**Authors:** T. X. R. Souza, C. A. Macedo

**Affiliations:** Departamento de Fisica, Universidade Federal de Sergipe, 49100-000 Sao Cristovao, SE, Brazil; Argonne National Laboratory, UNITED STATES

## Abstract

In this study, the ground state energies of face-centered cubic Hubbard clusters are analyzed using the Lanczos method. Examination of the ground state energy as a function of the number of particle per site *n* showed an energy minimum for face-centered cubic structures. This energy minimum decreased in *n* with increasing coulombic interaction parameter *U*. We found that the ground state energy had a minimum at *n* = 0.6, when *U* = 3*W*, where *W* denotes the non-interacting energy bandwidth and the face-centered cubic structure was ferromagnetic. These results, when compared with the properties of nickel, shows strong similarity with other finite temperature analyses in the literature and supports the Hirsh’s conjecture that the interatomic direct exchange interaction dominates in driving the system into a ferromagnetic phase.

## Introduction

The Hubbard model [1] is the most powerful model for studying the strongly correlated electrons in transition metals. In some of these metals the *d*-orbitals are only partially filled, and in the ferromagnetic state, the magnetic moment per atom is a fraction of the Bohr magneton (*μ*_*B*_). This behavior shows a significant itinerancy of *d*-band electrons, once the electron itinerancy may lead to a non-integer average number of electrons, which lead to the magnetic moment behavior cited above. The transition metals have narrow energy bands owing to the degeneracy of their d-orbitals and it is possible to use a single-band Hubbard model to study them. This model has two contributing terms: the first one represents electronic hopping, commonly considered only between the nearest neighbors, and the second term represents the intra-atomic coulombic interaction. The Hamiltonian for such a system is

$$\begin{array}{c} H=-t\sum_{i,j,\sigma }c_{i,\sigma }^{†}c_{j,\sigma }+U\sum_{i}n_{i,↑}n_{i,↓}, \end{array}$$ (1)

where *t* is the hopping integral, *U* represents the coulomb interaction potential energy, $c_{i,\sigma }^{†}$ (*c*_*i*,*σ*_) is the fermionic operator which creates (destroys) an electron with spin *σ* at site *i*, and *n*_*i*,↑_ is the particle number operator at site *i*.

Despite the simplicity of the model, full exact solutions in the thermodynamic limit have been obtained only for the one-dimensional structures [2–4]. In systems with half-filled bands, the ground state shows no long-range ferromagnetic correlation, and the system becomes insulating at *U* > 0. An interesting result was demonstrated by Ghosh [5], who showed that there is no ferromagnetism or antiferromagnetism in one- and two-dimensional Hubbard models at non-zero temperatures.

Finding solutions for higher dimensions remains a challenging task and many approaches have been put forward to study specific features and interaction scenarios using the Hubbard model including the mean-field approximation, perturbation theory, and dynamical mean-field approximation (DMFA). A more convenient approach relies on dealing with the model outside the thermodynamic limit, where it is possible to apply computational techniques such as exact diagonalization and quantum Monte Carlo (QMC) methods, which are discussed in this paper.

The electronic correlations among electrons present in the 3*d* band are crucial to understanding the magnetic properties of some transition metals such as Fe, Ni, and Co. The above-mentioned electronic itinerancy leads to magnetization values that are fractions of the Bohr magneton, which is valid for these three metals [6]. Nickel has a face-centered cubic (fcc) structure, and the ground state is ferromagnetic with a magnetic moment per atom of approximately 0.6*μ*_*B*_. This magnetic moment represents itinerant particles density, which in this case are roles rather than electrons [7, 8].

## Theoretical Method and Discussion

Several works have used the single-band Hubbard model [4, 5, 7, 8], however, an important development was made by Hirsch [9], who analyzed the intra-atomic interaction as a function of the interatomic direct exchange interaction and showed that the latter is the dominant in driving the system into a ferromagnetic phase. This conjecture was used to analyze the results from Ref. [7] and was also taken into account in this work. In Ref. [7], the fcc lattice was studied using the QMC simulation; however, the model did not perform well in the low-temperature regime. Hence, this work aims to analyze the ground-state properties of some fcc lattice clusters through a numerical diagonalization technique known as the Lanczos method [10]. The cluster structures were obtained by increasing the number of atoms in the fcc lattice structure along with their symmetries in order to maximize the number of bonds [8]. In Ref. [8], a small number of sites (*N*_*s*_), around 4–6, were considered because of computing limitations at that time. In this work, we were able to study 8–12 sites using new computer configurations and optimized Lanczos algorithm.

A set of orthogonal basis vectors was constructed for the Hamiltonian [Eq (1)] such that the matrix representation had a tridiagonal form. The algorithm was initiated with a random initial vector |*ϕ*_0_〉, from which a set of orthogonal vectors was spanned following the rule

$$\begin{array}{c} |\phi_{n+1}\rangle =H|\phi_{n}\rangle +a_{n}|\phi_{n}\rangle -b_{n}^{2}\left|\phi_{n-1}\right\rangle , \end{array}$$ (2)

where

$$\begin{array}{c} a_{n}=\frac{\left\langle \phi_{n}|H|\phi_{n}\right\rangle }{\left\langle \phi_{n}|\phi_{n}\right\rangle }\;\text{and}\;b_{n}^{2}=\frac{\left\langle \phi_{n}|\phi_{n}\right\rangle }{\left\langle \phi_{n-1}|\phi_{n-1}\right\rangle }. \end{array}$$ (3)

This resulted in the corresponding matrix representation of the Hamiltonian tridiagonal

$$\begin{array}{c} H_{T}=\left(\begin{array}{ccccc} a_{0} & b_{1} & 0 & \cdots & 0\\ b_{1} & a_{1} & b_{2} & \ddots & \vdots \\ 0 & b_{2} & a_{2} & \ddots & 0\\ \vdots & \ddots & \ddots & \ddots & b_{n-1}\\ 0 & \cdots & 0 & b_{n-1} & a_{n-1} \end{array}\right). \end{array}$$ (4)

The numerical instabilities in some systems require other approaches to the Lanczos method [11–13]. In this work, we used a modified explicitly restarted Lanczos method [13], in which the total spin of the system was used to analyze the ground state convergence. Without this approach, the Lanczos method failed to converge successfully for some of the systems analyzed in this work.

**Fig 1:**
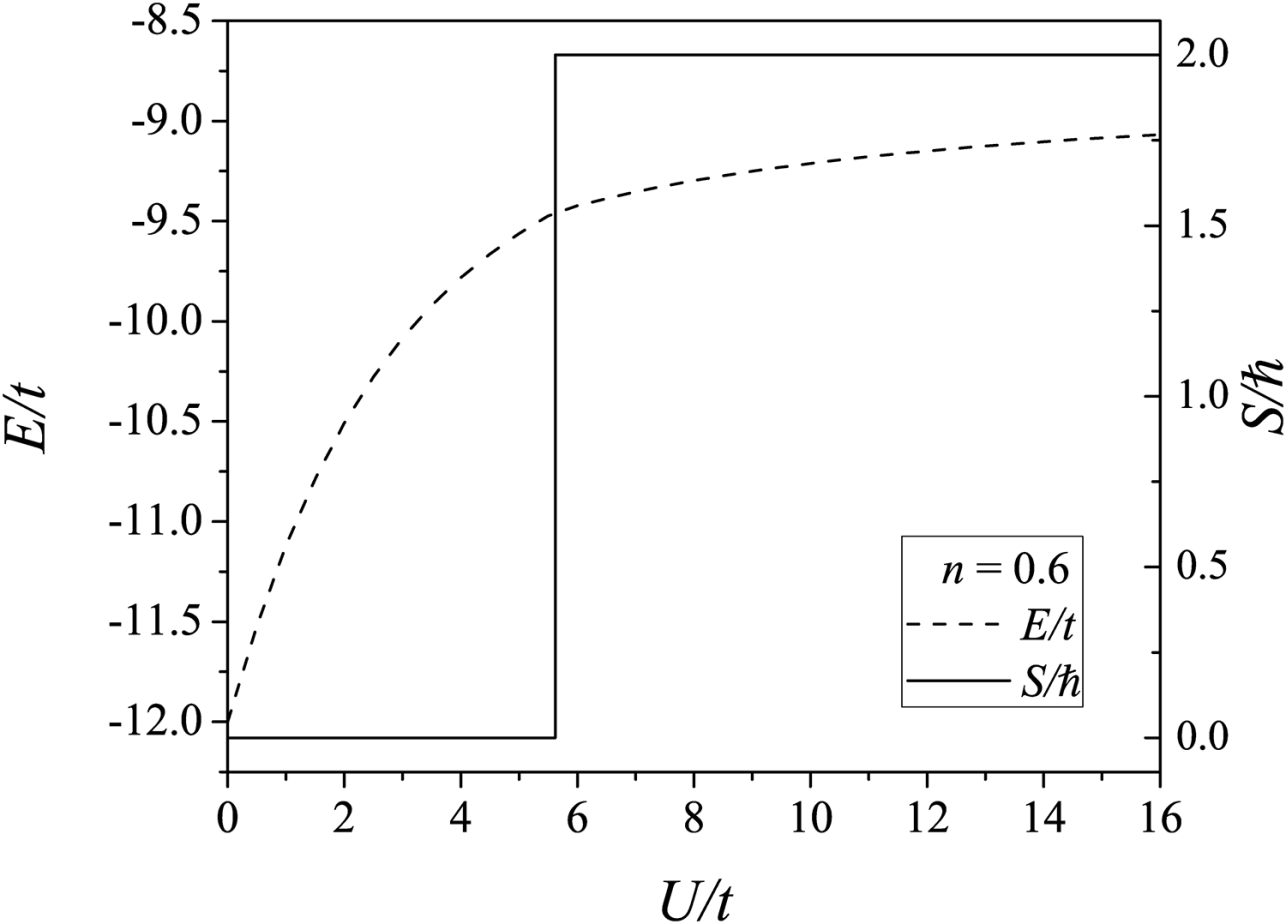
Ground state energy (dashed line) and total spin (solid line) of a fcc cluster as a function of *U*/*t* for a system with *N*_*s*_ = 10 and *n* = 0.6.

We initially determined the ground-state energy and total spin as a function of the coulombic interaction for the *n* = 0.6 (10 sites with six particles). Fig 1 shows a quantum-state transition near *U*/*t* = 5.62. At this point, the total spin of the system changes from 0 to 2, which indicates a shift in the magnetic correlation from paramagnetic to ferromagnetic.

**Fig 2:**
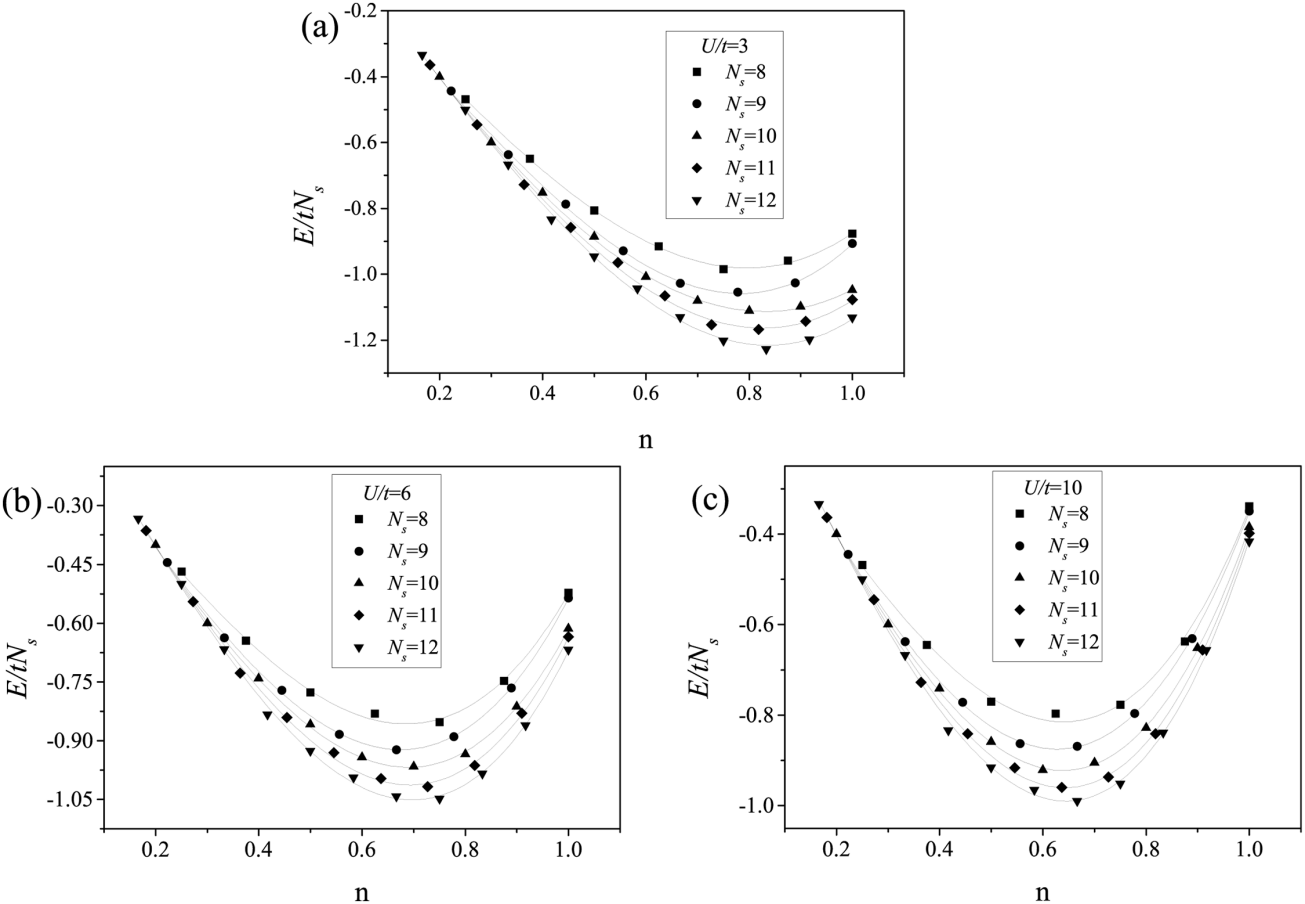
Ground state energy per site of an fcc cluster as a function of the particle density *n* with (a) *U*/*t* = 3, (b) *U*/*t* = 6, (c) *U*/*t* = 10 and number of sites varies from 8 to 12. The solid lines are guides for the eye.

The ground-state energy per site as a function of the particle density was then evaluated above the transition point (with the system in the ferromagnetic state) for *N*_*s*_ = 8, 9, 10, 11 and 12. When *U*/*t* = 10 (Fig 2(c)), we found that the energy reached a minimum at *n* ≈ 0.64, for all values of *N*_*s*_.

**Fig 3:**
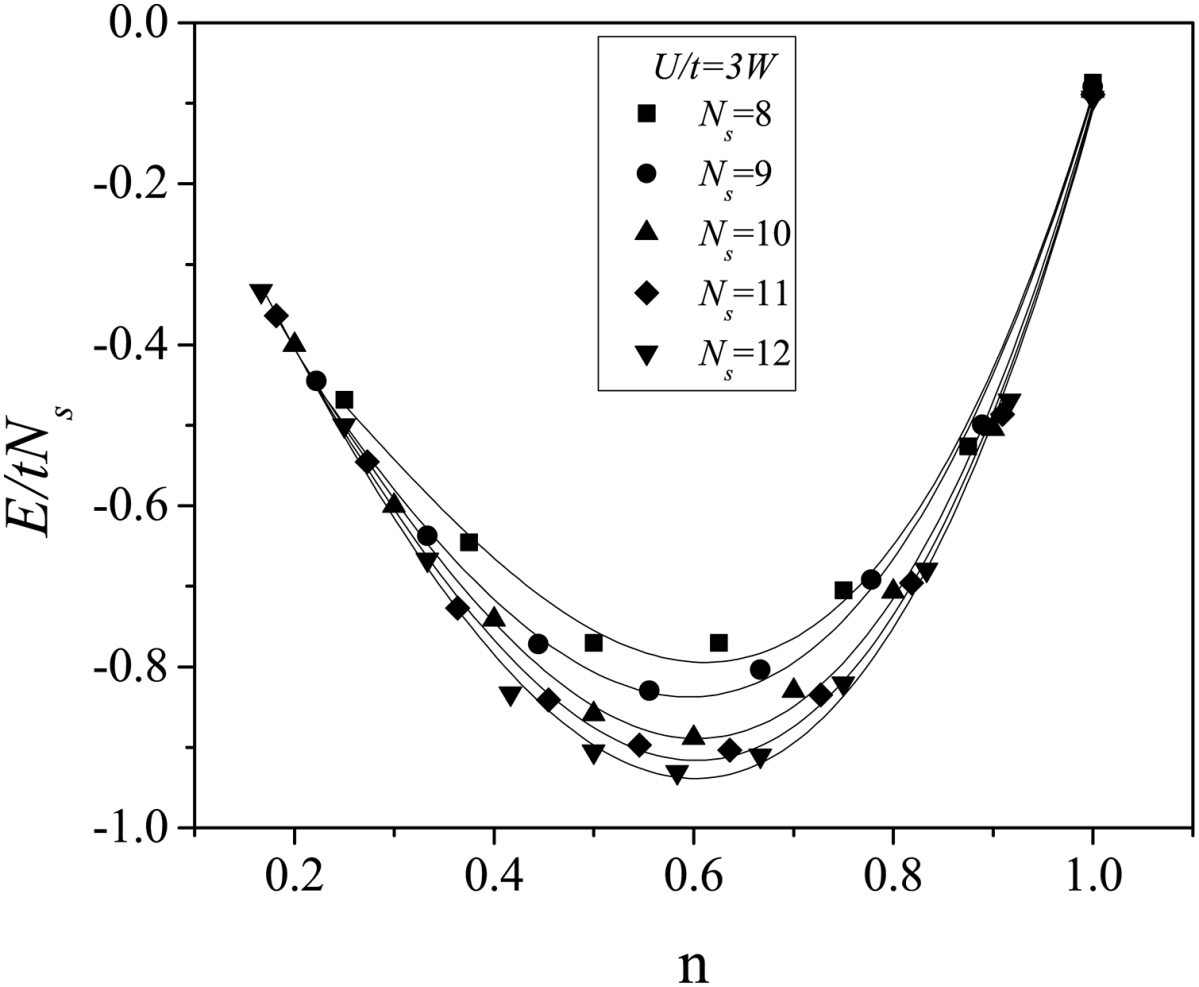
Ground-state energy per site for an fcc cluster as a function of the particle density *n* for *U*/*t* = 3*W*. The number of sites *N*_*s*_ varies from 8 to 12. The solid lines are guides for the eye.

Slightly above the transition point at *U*/*t* = 6 (Fig 2(b)), the energy minimum was found at *n* ≈ 0.69 for all values of *N*_*s*_. The increase in the *n* value with decreasing U/t shows the importance of coulombic interactions in the ferromagnetic state. The (Fig 2(a) reinforces this conclusion. It is also emphasizes the importance of the Hirsch conjecture, which includes additional electronic interactions [7, 9]. In Ref. [14] the DMFA was applied using an interaction parameter proportional to the non-interacting bandwidth (*W*), which for the fcc lattice is *W* = 16*t*. Ferromagnetism was found for *n* = 0.6 and *U* = 3*W*, and when this coulombic interaction parameter was used in the model of this work (Fig 3) the ground state energy minimum was located exactly at *n* = 0.6.

## Conclusion

We analyzed the electronic correlations in the fcc clusters using the single-band Hubbard model for several coulombic interaction regimes defined by *U*/*t*. For a fixed particle density of *n* = 0.6, we observed a quantum-state transition at *U*/*t* = 5.62, at which the total spin of the system changed from 0 to 2. For *U*/*t* = 3*W*, where *W* is the non-interacting energy bandwidth, the ground-state energy as a function of the particle density reached a minimum value at *n* = 0.6.

These results reinforce the analysis reported by Macedo and Souza [7] that was obtained using the QMC methods, which have serious limitations for analysis in low-temperature regimes. In this regime, the system shows a minimum ground state energy per site for higher *U*/*t* values than previously determined by the QMC methods.

When considering the properties of Ni, the *U*/*t* value that led to the lowest ground state energy at *n* = 0.6 was high. This highlights the importance of coulombic interactions in defining the electronic correlations that drive the ferromagnetic behavior in the fcc lattices. Furthermore, it indicates the need for an extended Hubbard model involving additional interaction parameters, as posited by Hirsch [9].
